# Neprilysin inhibition does not alter dynamic of proenkephalin‐A 119‐159 and pro‐substance P in heart failure

**DOI:** 10.1002/ehf2.13278

**Published:** 2021-03-20

**Authors:** Henrike Arfsten, Georg Goliasch, Philipp E. Bartko, Suriya Prausmüller, Georg Spinka, Anna Cho, Johannes Novak, Julia Mascherbauer, Helmuth Haslacher, Guido Strunk, Martin Hülsmann, Noemi Pavo

**Affiliations:** ^1^ Department of Internal Medicine II, Division of Cardiology Medical University of Vienna Waehringer Guertel 18‐20 Vienna 1090 Austria; ^2^ Department of Medical and Chemical Laboratory Diagnostics Medical University of Vienna Waehringer Guertel 18‐20 Vienna 1090 Austria; ^3^ Complexity Research Schönbrunner Straße 32 Vienna 1050 Austria

**Keywords:** Neprilysin, Substance P, Enkephalin, ARNi, Heart failure

## Abstract

**Aims:**

As NEP degrades many substrates, the specific therapeutic mechanism of NEP inhibition with angiotensin receptor neprilysin inhibitor (ARNi) in heart failure with reduced ejection fraction (HFrEF) is not entirely evident. The aim of this study was to investigate the response of two substrates of NEP—the tachykinin and enkephalin systems—to the initiation of ARNi therapy in HFrEF.

**Methods and results:**

Between 2016 and 2018, 141 consecutive patients with stable HFrEF [74 with initiation of ARNi and 67 controls on continuous angiotensin converting enzyme inhibitor (ACEi) or angiotensin receptor blocker (ARB) therapy] were prospectively enrolled. Plasma proenkephalin‐A 119‐159 (PENK) and pro‐substance P (pro‐SP) were serially determined. Proenkephalin‐A 119‐159 and pro‐SP correlated strongly with each other (*r_s_* = 0.67, *P* < 0.001) and kidney function (*r_s_* = −0.66, *P* < 0.001 and *r_s_* = −0.54, *P* < 0.001) and modestly with NT‐proBNP (*r*_*s*_ = 0.32, *P* < 0.001 and *r*_*s*_ = 0.24, *P* = 0.006, respectively). Concentrations of circulating PENK were slightly elevated after 1 and 2 year follow‐up compared with baseline (BL) [BL median: 67.4 pmol/L (IQR: 57.3–89.8), 1 year: 83.5 pmol/L (IQR: 62.4–111.6), 2 years: 92.3 pmol/L (IQR: 63.1–101.9); BL vs. 1 year: *P* = 0.017 and BL vs. 2 years: *P* = 0.019] in the overall analysis, but lost significance at 2 year follow‐up when assessed in paired subanalysis (*P* = 0.116). Plasma pro‐SP levels remained comparable during the entire follow‐up [BL median: 78.3 pmol/L (IQR: 67.9–90.6), 1 year: 75.9 pmol/L (IQR: 58.6–96.3), 2 years: 79.7 pmol/L (IQR: 59.9–105.3); *P* = ns for both timepoints]. Biomarker patterns of ARNi patients were independent from baseline therapy, that is, ACEi or ARB (*P* > 0.05 between groups).

**Conclusions:**

Although enkephalins and SP are known substrates of NEP, NEP inhibition by ARNi does not clearly affect the circulating precursors PENK and pro‐SP in HFrEF.

## Introduction

Sacubitril/valsartan is indicated for the treatment of heart failure with reduced ejection fraction (HFrEF).^1^, ^2^, ^3^ The compound acts by inhibition of neprilysin (NEP) and suppression of the renin‐angiotensin‐system (RAS). The key advantage of NEP inhibition (NEPi) has been perceived to be its ability to inhibit B‐type natriuretic peptide (BNP) breakdown thereby augmenting BNP actions. However, NEP is a promiscuous enzyme, involved in the degradation of numerous substrates. Enkephalin and substance P are among the substrates of NEP.^4^ It has been shown that these peptides of the endogenous opioid‐system and tachykinin‐system are expressed in the heart, containing local and systemic regulatory properties, and that plasma levels are increased in HFrEF^5^, ^6^ which justifies investigation in the context of NEPi.

The endogenous opioid system plays an essential role in cardioprotection and is activated in heart failure (HF).^5^ Proenkephalin‐A 119‐159 (PENK) represents a stable surrogate marker for active enkephalin which acts as an endogenous opioid.^5^ Plasma levels of PENK are associated with death and development of HF after myocardial infarction (MI) and adverse outcome in stable HF patients.^5^, ^7^

The effects of the vasoactive neuropeptide substance P (SP) of the tachykinin family on the heart are ambiguous including coronary vasodilation and increase in cardiac output but also inflammation and fibrosis.^8^ Elevated plasma pro‐SP, a stable surrogate marker for labile SP, was found to be prognostic for death and recurrent myocardial infarction (MI) in acute myocardial infarction patients.^7^

Due to the complexity of heart failure and the knowledge of multiple neuroregulatory peptides involved, which are also substrates of NEP, it is unlikely that the profound benefit of sacubitril/valsartan can be explained by its effects on natriuretic peptides solely.

Our study aimed to investigate short‐term and long‐term dynamic of the tachykinin‐system and opioid‐system, assessed by plasma pro‐SP and PENK levels as surrogates, after ARNi therapy initiation in HFrEF patients. We intended to extend the knowledge on so far unrecognized alternative biological mechanisms, potentially contributing to the therapeutic effects of NEPi.

## Materials and methods

### Study population

This is a non‐randomized observational two‐arm study (ARNi vs. ACE/ARB only). Patients with stable chronic HF with reduced ejection fraction (HFrEF)^1^ were consecutively enrolled from a prospective registry linked to a biobank at the heart failure clinic of the Vienna General Hospital, a university‐affiliated tertiary centre.

The study population and the protocol for biobank collection have been reported previously.^9^ Inclusion of patients in whom ARNi therapy was initiated, ranged between February 2016 and November 2018. The decision for the initiation of ARNi therapy was made according to the European Society of Cardiology heart failure guidelines and the treating physicians discretion.^1^ ARNi therapy was up‐titrated to the maximum tolerated dose within 2 weeks. These patients had to have a baseline blood sample and at least one available blood sample within 18 months after the initiation of ARNi. Patients on continuous therapy with angiotensin‐converting enzyme inhibitor (ACEi) or angiotensin receptor blocker (ARB) were enrolled as controls between February 2016 and June 2017. Control patients had to have baseline as well as 1 and 2 year follow‐up blood samples as defined later.

Comorbidities, traditional risk factors and medical therapy were recorded. Patients were followed‐up as clinically appropriate. Written informed consent was obtained from all study participants. The study protocol was approved by the ethics committee of the Medical University of Vienna and complies with the Declaration of Helsinki.

### Biobank and follow‐up intervals

Within the registry, serial venous blood sampling was performed routinely. For patients with therapy switch to ARNi, baseline samples were defined as samples obtained at the day of initiation or ARNi therapy. Short‐term follow‐up was defined as the first sample obtained after 4 weeks of therapy switch but not later than 6 months, 1 and 2 year follow‐up samples were defined as samples obtained at 12 ± 6 months and 24 ± 6 months, but closest to the follow‐up time of 12 and 24 months if more than one sample was accessible. Control patients with continuous ACEi/ARB were consecutively enrolled within the time period before the comprehensive implementation of ARNI to avoid a selection bias. Baseline samples were selected as the earliest samples available within the registry after stable target dosage of RASi was achieved.

### Laboratory analysis and biomarker assays

Routinely available measurements were performed according to the local laboratory's standard procedure.

Venous blood samples were immediately centrifuged, frozen and stored at −80°C until further use. Biomarker assays have been established and described previously and were performed by independent researchers in a blinded fashion.

Circulating PENK was determined by a chemiluminescence immunoassay (sphingotest® penKid® Sphingotec GmbH, Hennigsdorf, Germany) as described previously.^7^ The kit was established in a healthy donor cohort with a median of 58.7 pmol/L (34.6–106.0 pmol/L), and a central 95th percentile reference interval of 36–98 pmol/L.^10^ The lower detection limit was 5.5 pmol/L. Intra‐assay and interassay coefficients of variation were 6.4% and 9.5% at 50 pmol/L, and 4.0% and 6.5% at 150 pmol/L, respectively. In a reference population the mean ± SD was 46.6 ± 14.1 pmol/L, the median was 45 [range 9–518] pmol/L, and the 99th percentile was 80 pmol/L.^11^

The measurement of stable plasma pro‐SP has been performed by a specific immunoassay (Sphingotec GmbH, Hennigsdorf, Germany) as reported earlier.^7^, ^12^ The lower detection limit of the immunoassay was 2.3 pmol/L. The original pro‐SP assay has been established in 50 healthy donors yielding concentrations between 2.7 and 14.1 pmol/L (median 5.5 pmol/L).^12^ The modified assay applied in this analysis was used in 1148 acute myocardial infarction patients resulting in pro‐SP levels of (mean ± SD) 77.2 ± 55.7 pmol/L.^7^

B‐type natriuretic peptide was measured using the SIEMENS Advia Centaur assay. Reference levels of the assay were reported for 983 apparently healthy adults.^13^ Median BNP was 25.4 pg/mL (IQR 23.4–27.5). BNP showed a gradual increase with age and female sex^13^ and markedly higher levels in heart failure patients.^13^ Cut‐offs for the diagnosis of acute or chronic heart failure were derived from other landmark studies.^14^

As in PARADIGM‐HF,^15^ NT‐proBNP was measured with the Elecsys System 2010 by Roche Diagnostics (Mannheim Germany). Reference limits for the assay have been derived from the Framingham Heart Study cohort resulting in medians of 10.5–24.0 pg/mL for men and 34.2–57.7 pg/mL for women and the upper reference values (97.5th quantile) of 42.5–106.4 pg/mL in men and 111.0 to 215.9 pg/mL in women (depending on age).^16^

### Study outcome measures

Plasma concentrations of pro‐SP and PENK between baseline and follow‐up samples were compared.

### Data and statistical analysis

Discrete data are presented as count and percentage and analysed by using a *χ* ^2^ test. Continuous data are presented as median and interquartile range (IQR) and compared by using the Kruskal–Wallis test. The Spearman‐Rho correlation coefficient (*r_s_*) was calculated for PENK, pro‐SP, and laboratory parameters and clinical variables. Biomarker levels between different timepoints were compared using the Kruskal–Wallis and the Mann–Whitney *U*‐test. The Wilcoxon test was performed for paired analysis. For all tests two‐sided *P*‐values lower 0.05 were considered to indicate statistical significance. The statistical analysis was carried out with SPSS® software for Mac OS 10 operating system, Version 24.

## Results

### Baseline characteristics

Seventy‐four patients with therapy switch to ARNi and 67 control patients on continuous ACEi/ARB therapy were prospectively enrolled. Baseline characteristics including age, gender, co‐morbidities, medication and laboratory parameter levels were comparable between the ARNi patients and the control group, except for lower NYHA class and more frequent diagnosis of hypertension. Detailed baseline characteristics of the entire study population are displayed in *Table* *1*. Short‐term follow‐up after initiation of ARNi therapy was at a median of 86 days (IQR: 46–119) with samples of 65 patients available. One year follow‐up samples were obtained at a median of 359 days (IQR: 246–418; *n* = 53) and 2 year follow‐up samples at a median of 639 days (IQR: 615–726; *n* = 25) after therapy switch. Median time‐interval from baseline to 1 and 2 year follow‐up for control samples was 370 days (IQR: 301–392, *n* = 67) and 722 days (IQR: 640–742, *n* = 67), respectively.

**Table 1:** Baseline characteristics of the HFrEF patient cohorts undergoing therapy initiation with ARNi (*n* = 74) and on continuous therapy with ACEi/ARB (*n* = 67)

	ARNi switch (*n* = 74)	Continuous ACEi/ARB (*n* = 67)	*P*‐value
*General*			
Age, years (IQR)	62 (52–72)	66 (56–73)	0.215
Male gender, *n* (%)	54 (73)	53 (79)	0.552
BMI, kg/m^2^, (IQR)	26.6 (23.4–30.4)	28.4 (24.3–32.0)	0.192
Systolic BP, mmHg (IQR)	120 (115–135)	135 (120–150)	**0.003**
HR, b.p.m. (IQR)	68 (63–74)	69 (62–77)	0.887
NYHA class II/III, *n* (%)	48 (65) /26 (35)	34 (52) /15 (22)	**<0.001**
eGFR MDRD, mL/min/1.73 m^2^ (IQR)	70.6 (56.0–78.7)	59.6 (41.3–81.7)	0.056
NT‐proBNP, ng/L (IQR)	1872 (894–3079)	1468 (777–3219)	0.386
*Co‐morbidities*			
Known CAD, *n* (%)	40 (54)	31 (46)	0.401
Arterial hypertension, *n* (%)	38 (51)	46 (69)	**0.038**
T2DM, *n* (%)	19 (26)	21 (31)	0.458
Atrial fibrillation, *n* (%)	21 (28)	37 (46)	**0.002**
*Medication*			
BB, *n* (%)	71 (96)	65 (97)	1.000
ACEi/ARB/dual therapy, *n* (%)	47 (64) /23 (31%) /4 (5%)	45 (67) /20 (30) /2 (3)	0.751
MRA, *n* (%)	63 (85)	48 (72)	0.086
Ivabradine, *n* (%)	11 (15)	1 (1)	0.286
Furosemide, *n* (%)	29 (39)	25 (37)	1.000

Continuous variables are given as medians and IQR, counts are given as numbers and percentages.

ACEi, angiotensin‐converting enzyme inhibitor; ARB, angiotensin receptor blocker; ARNi, angiotensin receptor‐neprilysin inhibitor; BB, beta blocker; BMI, body mass index, BP, blood pressure; CAD, coronary artery disease; CRT, cardiac resynchronization therapy; HFrEF ‐ heart failure with reduced ejection fraction; HR, heart rate; ICD, inter cardiac defibrillator; IQR, interquartile range; MRA, mineralocorticoid receptor antagonist; NYHA, New York Heart Association; PM, pacemaker; T2DM, type 2 diabetes mellitus.

The median overall follow‐up time was 695 days (IQR: 443–831, range 150–1178) for patients with ARNi therapy and 941 days (IQR: 863–1081, range 658–1165) for the control group, that is, patients on ACEi/ARB therapy. During this follow‐up period, seven patients died and five patients received heart transplantation in the ARNi group and 10 patients died and one patient received heart transplantation in the control group.

At baseline, biomarker levels of pro‐SP were comparable between the ARNi and the control groups, but PENK levels were significantly higher in the control group [ARNi group: median 67.4 pmol/L (IQR: 57.3–89.8) vs. control group: median 84.3 pmol/L (IQR: 62.4–118.3; *P* = 0.014)].

### Correlation of pro‐SP and PENK with each other, laboratory parameters

Correlations of the biomarkers were assessed for the total patient population at baseline (*n* = 141). PENK and pro‐SP displayed a strong correlation with each other (*r_s_* = 0.67, *P* < 0.001). Furthermore, PENK and pro‐SP correlated moderately with kidney function (eGFR) (*r_s_* = −0.66, *P* < 0.001 and *r_s_* = −0.54, *P* < 0.001, respectively) and N‐terminal‐proBNP (NT‐proBNP) (*r_s_* = 0.32, *P* < 0.001 and *r_s_* = 0.24, *P* = 0.006, respectively). However, no correlation with BNP or association with NYHA classification was observed.

When assessing correlation between NT‐proBNP and PENK for each therapeutic group (ARNi and ACEi/ARB) separately, results were significant for both groups at baseline (ARNi: *r_s_* = 0.28, *P* = 0.020; ACEi/ARB: *r_s_* = 0.42, *P* < 0.001). At long‐term follow‐up, the results lost the statistical significance in the ARNi group (*r_s_* = 0.32, *P* = 0.13) but remained virtually unchanged for the ACEi/ARB (*r_s_* = 0.45, *P* < 0.001). At baseline, correlation between pro‐SP and NT‐proBNP was not significant in the ACEi/ARB group (*r_s_* = 0.24, *P* = 0.07) but reached significance for both treatment groups at long‐term follow‐up (ARNi: *r_s_* = 0.41, *P* = 0.048; ACEi/ARB: *r_s_* = 0.41, *P* = 0.001, respectively).

Correlation between PENK and pro‐SP with eGFR remained virtually unchanged significant at baseline and long‐term follow‐up when assessed for each treatment group separately (ARNi or ACEi/ARB: *P* ≤ 0.035 for 1 and 2 years, respectively).

### Effect of angiotensin receptor neprilysin inhibitor therapy initiation on proenkephalin‐A 119‐159 and pro‐substance P

**Figure 1:**
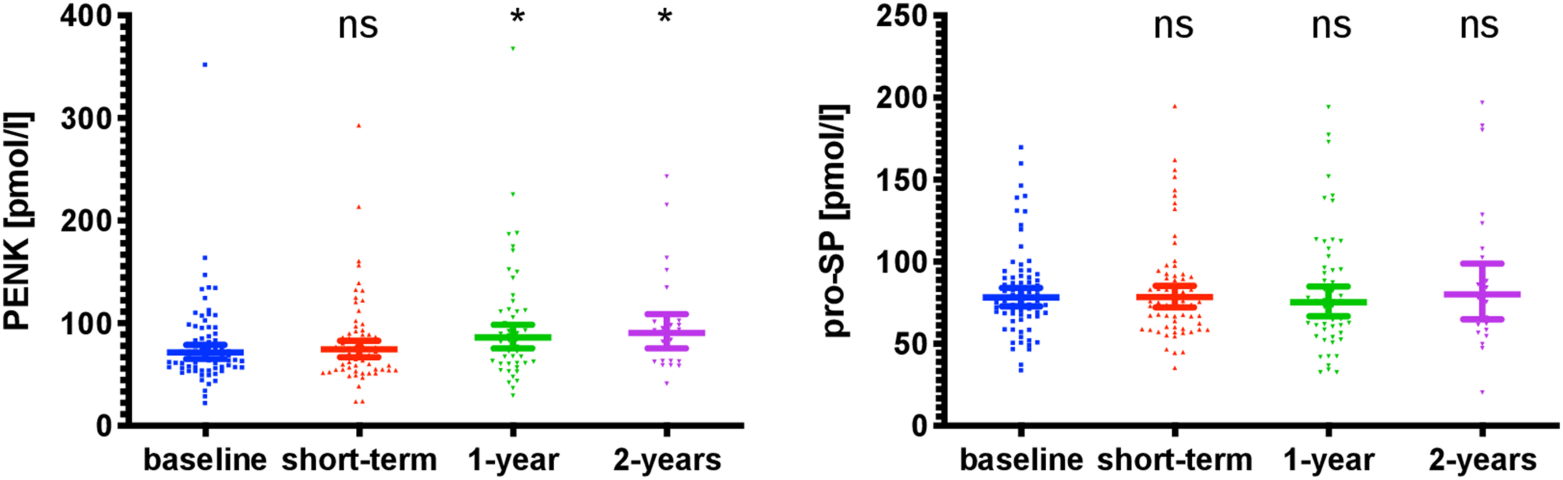
**Changes in plasma PENK and pro‐SP levels at short‐term, 1 and 2 year follow‐up after initiation of ARNi therapy.** Individual values as well as geometric mean and 95% CI of serum concentrations of biomarkers are displayed. Biomarkers were compared by the Mann–Whitney *U‐*test. ns for non‐significant with *P* ≥ 0.05, * for *P* < 0.05. CI, confidence interval; PENK, proenkephalin A 149; pro‐SP, pro‐substance P.

Plasma levels of hormones of interest following therapy switch to ARNi are displayed in *Figure* *1*. Concentrations of circulating PENK were slightly elevated after 1 and 2 year follow‐up [median 67.4 pmol/L (IQR: 57.3–89.8) vs. 83.5 pmol/L (IQR: 62.4–111.6), *P* = 0.017 and vs. 92.3 pmol/L (IQR: 63.1–101.9), *P* = 0.019]. However, when comparing concentrations at baseline and different follow‐up timepoints in a paired fashion for the respective patients with data available, differences at 2 year follow‐up lost significance [*n* = 25; median 65.0 pmol/L (IQR: 57.5–103.0) vs. 92.3 pmol/L (IQR: 63.1–101.9), *P* = 0.116]. Plasma pro‐SP levels remained comparable during the entire follow‐up [median 78.3 pmol/L (IQR: 67.9–90.6) vs. 1 year follow‐up: 75.9 pmol/L (IQR: 58.6–96.3), *p* = ns and vs. 2 year follow‐up: 79.7 pmol/L (IQR: 59.9–105.3), *P* = ns].

With respect, no changes in the assessed biomarkers occurred in the control group [PENK median 84.3 pmol/L (IQR: 62.4–118.3) vs. 1 year follow‐up: 82.5 pmol/L (IQR: 65.6–124.4), *P* = 0.608 and vs. 2 year follow‐up: 86.8 pmol/L (IQR: 59.6–125.1), *P* = 0.087; pro‐SP median 78.3 pmol/l (IQR: 67.9–90.6) vs. 1 year follow‐up: 77.6 pmol/L (IQR: 61.0–105.6), *P* = 0.757 and vs. 2 year follow‐up: 78.3 pmol/L (IQR: 65.6–101.1), *P* = 0.748].

The changes in NT‐proBNP and BNP levels have been described previously.^9^ A more detailed overview of assessed biomarker (PENK, pro‐SP; BNP, NT‐proBNP^9^) concentrations at baseline and different follow‐up timepoints for the respective patients is provided in *Table* *2*. Supporting Information, *Table* *S1* lists biomarker concentrations at baseline and different follow‐up timepoints for the respective patients with available data comparing medians by a paired test.

**Table 2:** Biomarker levels for HFrEF patients at baseline and after the initiation of ARNi as well as at short‐term and 1 and 2 years follow‐up

Therapy switch to ARNi					
Biomarkers	Baseline (*n* = 74)	Short‐term^a^ FUP (*n* = 65)	1 year^b^ FUP (*n* = 53)	2 years^c^ FUP (*n* = 25)	*P*‐value
PENK, pmol/L (IQR)	67.4 (57.3–89.8)	74.1 (54.9–89.9)	83.1 (62.4–111.6)	92.3 (63.1–101.9)	**0.021**
Pro‐SP, pmol/L (IQR)	78.3 (67.9–90.6)	75.4 (60.3–91.4)	75.9 (58.6–96.3)	79.7 (59.9–105.3)	0.879
BNP, pg/mL (IQR)	268.3 (117.2–487.4)	241.1 (96.3–591.8)	290.6 (105.6–575.5)	556.5 (171.1–850.3)	0.126
NT‐proBNP, pg/mL (IQR)	1872 (894–3079)	1152 (459–2441)	1166 (534–2490)	2267 (995–3295)	**0.036**
Continuous ACEi/ARB					

Biomarkers	Baseline (*n* = 67)	‐	1 year^d^ FUP (*n* = 66)	2 years^e^ FUP (*n* = 65)	*P*‐value
PENK, pmol/L (IQR)	84.3 (62.4–118.3)	‐	82.5 (65.6–124.4)	86.8 (59.6–125.1)	0.853
Pro‐SP, pmol/L (IQR)	78.0 (66.4–103.7)	‐	77.6 (61.0–105.6)	78.3 (65.6–101.1)	0.959
BNP, pg/mL (IQR)^a^	176 (95–327)	‐	156 (82–363)	160 (97–351)	0.905
NT‐proBNP, pg/mL (IQR)^a^	1468 (777–3219)	‐	1244 (568–2647)	1195 (567–3106)	0.683

ARNi, angiotensin receptor‐neprilysin inhibitor; BNP, B‐type natriuretic peptide; FUP, follow‐up; HFrEF, heart failure with reduced ejection fraction; IQR, interquartile range; NT‐proBNP, N‐terminal pro‐B‐type natriuretic peptide; pro‐SP, pro‐substance P; PENK, proenkephalin A 119‐159.

^a^ ARNi group: Short‐term FUP: median of 86 days (IQR: 46–119) after therapy switch.

^b^ ARNi group: 1 year FUP: median of 359 days (IQR: 246–418) after therapy switch.

^c^ ARNi group: 2 year FUP: median of 639 days (IQR: 615–726) after therapy switch.

^d^ Controls: 1 year FUP: median of 370 days (IQR: 301–392).

^e^ Controls: 2 year FUP: median of 722 days (IQR: 640–742).

**Figure 2:**
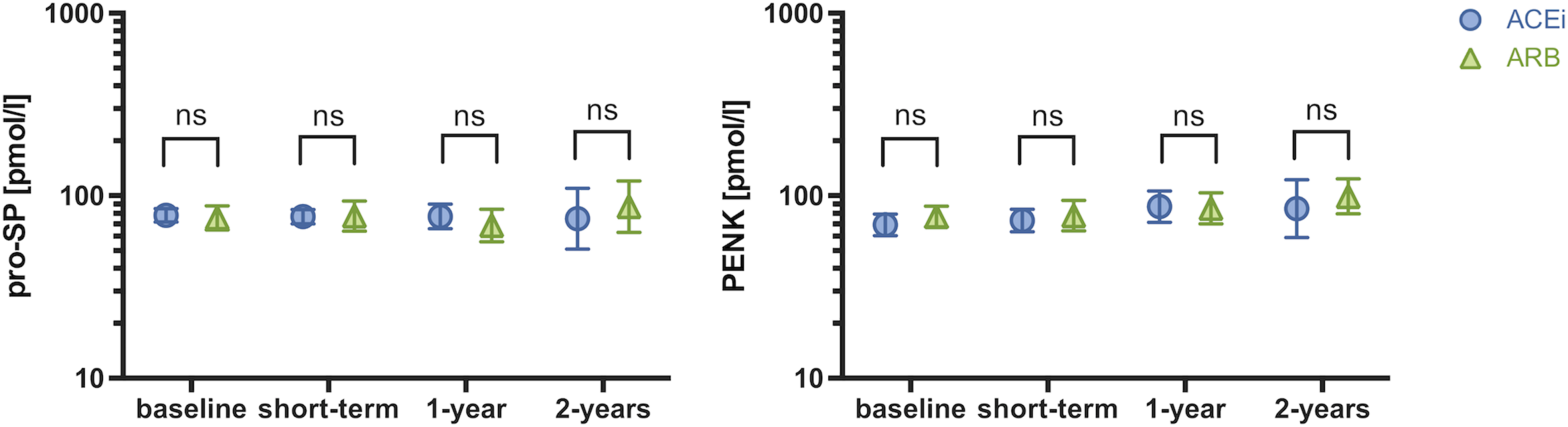
**Short‐term and long‐term changes in PENK and pro‐SP after the initiation of ARNi therapy according to the mode of RAS‐blockade at baseline.** Baseline data as well as short‐term and long‐term follow‐up levels are displayed as geometric mean and 95% CI. Group comparisons between different modes of RAS inhibition at baseline, that is, ACEi or ARB, were made by the Mann–Whitney *U*‐test. ns for non‐significant with *P* ≥ 0.05. ACEi, angiotensin‐converting enzyme inhibitor; ARB, angiotensin receptor blocker; PENK, proenkephalin A 119‐159; pro‐SP, pro‐substance P.

As depicted in *Figure* *2*, biomarker patterns for patients switched to ARNi were comparable and independent from therapy at baseline (*P* > 0.05 for all comparisons ARB vs. ACEi).

## Discussion

The distinct effect of ARNi on the dynamics of the endogenous opioid‐system and the tachykinin‐system in HFrEF have not been elucidated before. We demonstrated that PENK and pro‐SP correlate with heart failure severity reflected by NT‐proBNP levels. Both neurohormones correlated with kidney function in the overall HFrEF study‐population. However, while absolute and fold change of PENK transiently increased at 1 year follow‐up, the investigated blood levels of PENK and pro‐SP seem to be unaffected by NEPi at long‐term follow‐up after the initiation of ARNi therapy.

### Mechanism of action of neprilysin inhibition as the novel therapeutic approach in heart failure

The particular effects of ARNi, which are responsible for its remarkable clinical benefits,^2^, ^17^ remain a matter of debate. NEP is a transmembrane zink‐dependent metallopeptidase and is implicated in the degradation of numerous peptides with varying substrate selectivity.^18^ Several potential bioactive signalling pathway candidates have been identified including enkephalins and substance P.^19^

The primary hypothesis for the mechanism of action of NEPi in HFrEF specifically is to increase endogenous NP levels by blocking their inactivation.^18^, ^20^ A modest increase in BNP at short term after therapy initiation has been shown.^15^ The increase was accompanied by a decrease in its inactive cleavage product NT‐proBNP.^15^ However, BNP is a rather humble NEP substrate. Ad interim, first data on the effects of NEPi on further vasoactive NEP substrates with significance in HFrEF exist.^9^, ^20^, ^21^ For NEPi, a significant increase in atrial natriuretic peptide (ANP), substance P, adrenomedullin (ADM) and glucagon‐like peptide 1 (GLP‐1) at short‐term follow‐up of 90 days has been reported.^20^ Early dynamics in blood levels of these neurohormones after NEPi initiation, partly even exceeding the observed changes in BNP, substantiate the assumption of a more complex mechanism of action.

Earlier our group showed an ARNi mediated direct and indirect stable and substantial increase of the adrenomedullin‐axis at short‐term and long‐term follow‐up.^9^ ADM has a rather systemic than cardiac origin and is described as a marker of increased plasma volume and an activated sympathetic nervous system, both pathophysiologic conditions in imbalance characteristic for heart failure.^22^, ^23^

Because a potential influence on heart failure has also been described for enkephalins and SP, a further axis of action of these neurohormones would have been evident in NEPi and grounds the characterization of these neurohormones at least at the circulatory level.

### The enkephalin and tachykinin systems remain uninfluenced by angiotensin receptor neprilysin inhibitor therapy

#### Enkephalin system

Enkephalins and their receptors are produced in the central nervous system (CNS), the kidney, and the heart.^5^ Even though enkephalins exert regulatory effects in both the central and peripheral nervous system, most of the enkephalins in the heart originate from cardiomyocytes in response to stress and work autonomously in an autocrine or paracrine manner, independent of central activation.^24^, ^25^ Heart failure is characterized by compensatory sympathetic overstimulation to maintain cardiovascular homeostasis in response to reduced cardiac output. The initial beneficial compensatory increase in sympathetic neuronal activity with increased systemic vascular resistance, ventricular inotropy and increased heart rate, however, enhance the cardiac workload and afterload ceasing in a deteriorating vicious cycle over the long term. Enkephalins, co‐released with catecholamines in the heart, inhibits the cardiac sympathetic activation and vascular constriction.^26^ They have a cardio‐depressive effect, characterized by hypotension and bradycardia, negative inotropy and thus a protective reduction in cardiac oxygen demand by limiting work performance.^27^ Further potentially beneficial effects of enkephalins might be reasoned by influencing and opposing the neurohumoral activity, one of the most significant driving forces in heart failure.^28^

PENK is a byproduct of the cleavage of bioactive enkephalins from preproenkephalin A. Due to its high stability (48 h at room temperature) and stoichiometric generation with mature PENK‐A derived peptides it serves as a valid surrogate marker of enkephalins, which are susceptible to rapid enzymatic degradation.^5^ Previous research found elevated PENK levels to be associated with more advanced HF,^29^, ^30^, ^31^ and consistent with the direct cardio‐depressive effects of enkephalin as lower blood pressure and lower heart rate. Previous preliminary evidence of the association between circulating plasma Δα‐endorphin and the early clinical improvement after ARNi treatment has been reported.^21^

We found a significant increase in PENK levels at 1 year follow‐up after ANRi therapy initiation, that was not correlated to disease progression, reflected by NT‐proBNP. As enkephalins but not proenkephalins are among the identified NEP substrates, the observed increase in PENK might be indirectly triggered by NEPi. However, up to now, circulatory PENK levels are not proven to be representative for enkephalin concentration or activity. Secondary mediated causes for the observed PENK elevation cannot be excluded. Moreover, based on the limited number of patients analysed pairwise, the hereby presented observation requires cautious interpretation but provides potential for future analysis. Generally, opioid peptides are poorly studied in heart failure and no comparable data on NEPi exist. But linkage of the data available supports the suggestion of a heterogeneous and idiosyncratic response of endogenous opioids to NEPi.

#### Tachykinin‐systems (substance P)

Substance P belongs to the tachykinin family, is widely distributed in the CNS and the cardiovascular system and exerts its biological actions mainly via the neurokinin receptor 1 (NK1).^8^ In the heart, SP is predominantly found in the intrinsic cardiac nervous system^8^ stimulating parasympathetic components of the autonomic nervous system, with negative chronotropy and inotropy which consequently increases contraction forces.^32^ A protective role in the acute ischemia/reperfusion injury was attributed to SP.^33^ Best investigated are the neuropeptides strong vasodilatory effects on the coronary bed and the peripheral vasculature.^34^ Intravenous administration of SP results in decreased blood pressure.^35^ However, SP has double‐edged properties, stimulates cardiac fibrosis and activates the production of proinflammatory cytokines and adverse remodelling.^36^ Also, an enhanced SP mediated inflammation was reported in a translational NEPi model.^37^ For HF, NK1‐antagonists were proposed as a potential therapeutic option.^38^

Pro‐SP and SP are derived in a 1:1 ratio from the same precursor molecule pro‐tachykinin A.^12^ NEP is involved in the degradation of SP.^39^ Unlike our observation, an early increase of SP levels after the initiation of ARNi has been reported (determined by a commercial radioimmunoassay).^20^ When interpreting the results comparatively the different analytical methods applied must be noted. In our study, plasma pro‐SP levels remained unchanged after the initiation of ARNi indicating that NEPi has a neutral effect on pro‐SP concentration.

## Conclusions

Despite evidence indicating enkephalins and SP to be substrates of NEP, NEP inhibition by ARNi does not clearly affect long‐lasting changes of circulating prohormones PENK and pro‐SP in HFrEF. Based on the pilot nature of the presented data larger patient numbers are needed to confirm the presented results.

## Limitations

The study might be limited by the relatively small sample size, which however is also unique for patients with ARNi therapy in a single‐centre setting. Due to the nature of the study the reported findings are of preliminary nature and should stimulate further research and discussion about the mechanisms of action and the involvement of opioid‐system and tachykinin‐system in ARNi therapy and HFrEF. Measurement of the active form of both hormones would also give further insights about the clinical interdependency between ARNi, enkephalins, or SP, respectively.

## Conflict of interest

None declared.

## Supporting information


**Table S1.** Biomarker levels for HFrEF patients after the initiation of ARNi at short‐term and 1‐year and 2‐years follow‐up. Wilcoxon test was used for paired analysis.
