# Supplementary material for: Neprilysin inhibition does not alter dynamic of proenkephalin‐A 119‐159 and pro‐substance P in heart failure
Source: ESC Heart Fail. 2021 Mar 20;8(3):2016–24. doi: 10.1002/ehf2.13278 (PMC8120349; doi:10.1002/ehf2.13278)
Supplement: Supplementary file 1 — Table S1. Biomarker levels for HFrEF patients after the initiation of ARNi at short‐term and 1‐year and 2‐years follow‐up. Wilcoxon test was used for paired analysis. [file EHF2-8-2016-s001.doc]

**SUPPLEMENTARY MATERIAL**

**Supplementary Table 1. Biomarker levels for HFrEF patients after the initiation of ARNi at short-term and 1-year and 2-years follow-up.** Wilcoxon test was used for paired analysis.

|  | **Short-term FUP*(n=65)** | | | **1-year FUP^§^(n=53)** | | | **2-years FUP^¥^ (n=25)** | | | |  |
| --- | --- | --- | --- | --- | --- | --- | --- | --- | --- | --- | --- |
| Biomarkers | Baseline | FUP | p-value | Baseline | FUP | p-value | | Baseline | FUP | p-value | |
| PENK, pmol/l(IQR) | 65.0(57.3-85.0) | 74.1(54.9-89.9) | 0.268 | 63.3(56.4-85.0) | 83.1(62.4-111.6) | **<0.001** | | 65.0(57.5-103.0) | 92.3(63.1-101.9) | 0.116 | |
| Pro-SP, pmol/l(IQR) | 76.4(66.8-90.3) | 75.4(60.3-91.4) | 0.987 | 75.9(66.8-92.4) | 75.9(58.6-96.3) | 0.241 | | 82.6(67.9-99.9) | 79.7(59.9-105.3) | 0.668 | |
| BNP, pg/ml(IQR) | 243.6(116.5-578.6) | 241.1(96.3-591.8) | **0.049** | 293.9(124.8-464.2) | 290.6(105.6-575.5) | 0.152 | | 295.1(179.1-437.6) | 556.5(171.1-850.3) | **0.024** | |
| NT-proBNP, pg/ml(IQR) | 1867(868-3395) | 1152(459-2441) | **0.008** | 1882(1021-3063) | 1166(534-2490) | **0.005** | | 2055(1741-2295) | 2267(995-3295) | 0.459 | |

ARNi–angiotensin receptor-neprilysin inhibitor; FUP–follow-up; HFrEF–heart failure with reduced ejection fraction; IQR–interquartile range; pro-SP–pro-substance P; PENK–proenkephalin A 119-159; BNP–B-type natriuretic peptide; NT-proBNP–N-terminal pro-B-type natriuretic peptide.

*Short-term FUP: median of 86days (IQR:46-119) after therapy switch.

^§^1-year FUP: median of 359days (IQR:246-418) after therapy switch.

^¥^2-years FUP: median of 639days (IQR:615-726) after therapy switch.
